# Intensity standardisation of 7T MR images for intensity-based segmentation of the human hypothalamus

**DOI:** 10.1371/journal.pone.0173344

**Published:** 2017-03-02

**Authors:** Stephanie Schindler, Jan Schreiber, Pierre-Louis Bazin, Robert Trampel, Alfred Anwander, Stefan Geyer, Peter Schönknecht

**Affiliations:** 1 Department of Psychiatry and Psychotherapy, University Hospital Leipzig, Leipzig, Germany; 2 Department of Neurophysics, Max Planck Institute for Human Cognitive and Brain Sciences, Leipzig, Germany; 3 Department of Neuropsychology, Max Planck Institute for Human Cognitive and Brain Sciences, Leipzig, Germany; 4 Department of Neurology, Max Planck Institute for Human Cognitive and Brain Sciences, Leipzig, Germany; University of Minnesota, UNITED STATES

## Abstract

The high spatial resolution of 7T MRI enables us to identify subtle volume changes in brain structures, providing potential biomarkers of mental disorders. Most volumetric approaches require that similar intensity values represent similar tissue types across different persons. By applying colour-coding to T1-weighted MP2RAGE images, we found that the high measurement accuracy achieved by high-resolution imaging may be compromised by inter-individual variations in the image intensity. To address this issue, we analysed the performance of five intensity standardisation techniques in high-resolution T1-weighted MP2RAGE images. Twenty images with extreme intensities in the GM and WM were standardised to a representative reference image. We performed a multi-level evaluation with a focus on the hypothalamic region—analysing the intensity histograms as well as the actual MR images, and requiring that the correlation between the whole-brain tissue volumes and subject age be preserved during standardisation. The results were compared with T1 maps. Linear standardisation using subcortical ROIs of GM and WM provided good results for all evaluation criteria: it improved the histogram alignment within the ROIs and the average image intensity within the ROIs and the whole-brain GM and WM areas. This method reduced the inter-individual intensity variation of the hypothalamic boundary by more than half, outperforming all other methods, and kept the original correlation between the GM volume and subject age intact. Mixed results were obtained for the other four methods, which sometimes came at the expense of unwarranted changes in the age-related pattern of the GM volume. The mapping of the T1 relaxation time with the MP2RAGE sequence is advertised as being especially robust to bias field inhomogeneity. We found little evidence that substantiated the T1 map’s theoretical superiority over the T1-weighted images regarding the inter-individual image intensity homogeneity.

## Introduction

### Background

Dr David Kupfer, leading the revision of the world-wide used diagnostic system for mental diseases DSM IV [1], diagnosed “a failure of our neuroscience and biology to give us the level of diagnostic criteria, a level of sensitivity and specificity that we would be able to introduce into the diagnostic manual” [2]. The call for biological markers to replace self-report and exploration could hardly be made clearer. To this end, sub-millimetre resolution achieved by 7T magnetic resonance imaging (MRI) holds great potential because it opens the window to small volumes in candidate brain structures of psychiatric patients *in vivo*. To actually benefit from the high resolution of 7T MRI, post-processing and subsequent analysis need to be equally precise. In this context we previously established colour-coding as an important tool to ensure high reliability and reasonable time costs of computer-assisted segmentations of the hypothalamus on 7T T1-weighted MR images [3]. The hypothalamus is not only a relevant brain region in various psychiatric disorders (e.g. anxiety, depression, schizophrenia, and paedophilia) but it is also involved in neurodegenerative disorders (e.g. Huntington’s disease, Wernicke’s encephalopathy) and other conditions like obesity, narcolepsy, and migraine [4]. An adequate colour-coding enhances the visual contrast of its anatomical boundaries and helps the human eye to distinguish between relevant and non-relevant details. Like many methods analysing image intensities, however, colour-coding requires similar tissues to be displayed with similar intensities throughout one image and across different images. A number of factors can corrupt the image intensity causing intrascan intensity variation or interscan intensity variation [5, 6]. Most intrascan signal variations can be prevented by using modern MRI hardware and optimal acquisition parameters, but, at higher field strengths, increased bias field artefacts have to be addressed. There are a number of established post-processing algorithms for bias field correction (for reviews, see e.g. [7, 8]). Alternatively, the magnetisation-prepared 2 rapid acquisition gradient echoes sequence (MP2RAGE [9]) provides bias-field corrected images at high field strengths. Nonetheless, residual interscan intensity variation in the T1-weighted images of the MP2RAGE exists, as we will demonstrate by colour-coding. To correct such differences and thereby reduce erroneous variance in the resulting segmentation, the unique intensity scale of each image needs to be standardised.

### Related intensity standardisation techniques

#### Transformation functions

The basic principle of intensity standardisation is to replace the intensity values of a target image (input intensity) with new intensity values (output intensity) that are assigned by a transformation function. A model of the intensity relationship between the target and the reference image yields this transformation function. The simplest reasonable transformation would be to introduce an offset value (e.g. the mean intensity difference between both images) that corrects over- or underexposure of the entire target image. When the strength of over- or underexposure scales systematically with higher intensities, a linear model will give a more accurate description of the relationship between target and reference image intensities [5, 6, 10]. If there is cause to assume tissue-specific over- or underexposure [11], we can introduce break points at certain intensity levels (e.g. at tissue boundaries) and use a piecewise linear transformation function [12–15]. To correct more complex intensity patterns, non-linear models are required. This usually involves higher computational complexity and requires prior theoretical assumptions (e.g. the order of a fitted polynomial [16]), or additional information like multi-modal [17–19] or longitudinal [20] scan data.

#### Information input—Intensity domain

The transformation function is modelled by relating the target image intensities to those of the reference image. Either the image intensity information is evaluated alone, or in combination with the images’ spatial information.

Reducing the two images to their intensity information, namely, intensity histograms, which are then aligned, is generally called histogram matching. Replicating our systematisation above, we could simply start by correcting a global shift between the two histograms. By assigning, for example, the mean intensity of the reference histogram ${\bar{I}}_{R}$ to the mean intensity of the target histogram ${\bar{I}}_{T}$, and assuming ${\bar{I}}_{T}={\bar{I}}_{R}+n$, we can estimate the offset value *n* by which the target image is over- or underexposed compared with the reference image. With two or more definite assignments we can estimate a linear model with the scaling factor *m* by which the target histogram is stretched or compressed as compared with the reference histogram: ${\bar{I}}_{T}={\bar{I}}_{R}\cdot m+n$. Christensen [5] achieved reproducible corrections for T1-, T2-, and density-weighted images using the origin of the coordinate system as the first assignment (i.e. assuming a negligible offset value *n* = 0) and the white matter (WM) mode as the second assignment. Alternatively, the scaling factor can be estimated by minimising the squared differences between the two histograms [10].

For tissue-specific corrections, three or more assignments can be used to divide the intensity range into compartments with separate linear scaling factors for each intensity compartment, resulting in a piecewise linear transformation function: Nyúl and Udupa [12] initially matched the histograms’ global mode plus low- and high- percentile points. The intensities of the histogram modes of the reference image assigned to the intensities of the corresponding target histogram modes constitute the anchor points of the transformation function. By piecewise linear interpolation between the anchor points, the intensity levels of the reference image are assigned to those of the target image. As skull-stripped MP2RAGE images are consistently multimodal with distinct peaks corresponding to WM, grey matter (GM), and cerebrospinal fluid (CSF), additional percentile anchor points were dispensable in our variant of piecewise linear histogram matching (PHM).

As stated above, non-linear functions can be developed by introducing additional assumptions or information. Hellier [16] assumed normal distributions for the major tissue classes and approximated the image intensity histogram by modelling each tissue class with a Gaussian probability density function. A curve was then fitted to the tissue-specific means of the target and the reference image by minimising a cost function. Since the crucial assumption concerning the order of the fitted polynomial was not specified by the author, we developed an alternative, non-linear variant using the histogram modes from the PHM.

Comparing several density-, T1-, and T2-weighted sequences, Nyúl and colleagues [13] observed that the global mode of the intensity histogram might correspond to WM in one image but to GM in another image. To avoid the resulting tissue mixing, they recommended the sole use of histogram percentiles (median, quartiles, and deciles). However, even percentile matching has later been reported to mix tissue types in T1-weighted images or relative cerebral blood volume maps [15, 21]. This might be avoided by our third method—an extreme variant that matches all possible percentiles by matching the cumulative histogram (CHM). Here, *each* intensity level is interpreted as a separate compartment and is assigned (in this case we could say “replaced by”) the intensity level of the reference image with the most similar relative cumulative frequency [22–24]. The assumption underlying this non-linear standardisation is that matched cumulative intensity frequencies correspond to matched image intensities.

#### Information input—Intensity and spatial domain

Estimating the transformation function using only the intensity histograms assumes that images with similar histograms look more alike. If target and reference intensity distributions are likely divergent in truth (i.e. different heights and spread of the tissue histogram modes due to atrophy, different contrast), they should not be matched without restrictions. Such a restriction is spatial correspondence. It can be incorporated in intensity standardisation procedures by analysing intensity information tissue-wise or voxelwise.

Leung et al. [6] performed *k*-means clustering of T1-weighted images to extract the three major tissue types (WM, GM, and CSF) and applied linear regression between the corresponding tissue cluster means of the target and reference images to estimate a global linear scaling factor. Given our focus on the GM of the hypothalamus and surrounding WM, we opted for a linear standardisation using two precise regions of interest (ROIs) instead of tissue class maps. Cataldo et al. [15] argued for an atlas-driven tissue class segmentation prior to intensity standardisation. They applied Nyúl and Udupa’s decile variant separately to each tissue class to prevent the mixing of the major tissue types. Meier and Guttmann [11] approximated the histograms of automatically segmented tissues by Gaussian distributions. The estimated means and standard deviations (SD) of each tissue were then used to correct the brightness (matching the mean) and contrast (matching the SD) of each tissue by separate linear scaling factors. Put together, the hypothetical transformation function for the entire image would be a non-continuous piecewise linear mapping function. Closely related to these approaches is our segmentation-based piecewise linear standardisation (SPS).

Considerably more complex, but for the sake of completeness to be acknowledged here as voxelwise approach, is the matching of joint histograms. Each point in a joint intensity histogram represents by its colour or shading the probability of a certain intensity combination for spatially corresponding voxels of two images. They can be obtained from images of different subjects, modalities (e.g. T1 versus T2), or successive scans and have been successfully applied in conjunction with prior tissue segmentation and non-rigid image registration [14, 17–20].

In sum, as the sources of the interscan intensity variation in our data were unknown, we chose to compare very different approaches, ranging from global linear models to piecewise linear models, up to non-linear models and T1 mapping, using either intensity information alone or incorporating both intensity and spatial information. Methods with reasonable simplicity and practicability were preferred.

## Methods

### Image acquisition and pre-processing

MRI was performed with a 7T whole-body scanner (MAGNETOM 7T, Siemens, Erlangen, Germany) and a 24-channel NOVA coil (Nova Medical, Inc., Wilmington MA, USA). A 3D MP2RAGE [9] was used with repetition time (TR) = 8250 ms; inversion times (TI1/TI2) = 1000/3300 ms; flip angles (FA1/FA2) = 7°/5°; echo time (TE) = 2.51 ms; bandwidth (BW) = 240 Hz/Px, 1 average. A field of view (FOV) of 224 mm x 224 mm x 168 mm and an imaging matrix of 320 x 320 x 240 resulted in a nominal acquisition voxel size of 0.7 mm isotropic. Parallel imaging (GRAPPA [25]) was used with an acceleration factor of 2, achieving a scan time of 18:02 min. The uniform MP2RAGE images, referred to as T1-weighted images in this text, and the quantitative T1 maps were skull-stripped using Medical Image Processing and Visualization (MIPAV) software (version 7.0.1 [26]) with the CBS High-Res Brain Processing Tools 3.0 [27].

### Sample selection procedure

Data from 84 subjects (51 women; 33 men; mean age ± SD: 39 years ± 13 years) without neurological diseases were available. All subjects had given written informed consent and the study was approved by the Ethics Committee of the University of Leipzig. The native T1-weighted images were segmented into the three major tissue classes (WM, GM, and CSF) with the MIPAV module for the Fuzzy And Noise Tolerant Adaptive Segmentation Method (FANTASM [28]). The resulting hard segmentation map was used to identify GM and WM voxels. The median intensities of the GM voxels (sample mean = 1907 ± 76) and the WM voxels (sample mean = 3246 ± 58) were estimated, and a reference image was chosen with representative GM and WM median intensities (female, 56 years). Twenty images with very dark or bright GM and/or WM, that is, with median intensities within the outermost deciles of the sample, were chosen as target images. They belonged to 9 women and 11 men with a mean age of 40 years ±12 years.

### Intensity standardisation procedures

#### Piecewise Linear Histogram Matching (PHM)

Intensity histograms of skull-stripped MP2RAGE brain images are multimodal, with distinct peaks corresponding to the three major tissue types. By estimating local peaks in defined intensity windows, we can ensure correct tissue correspondence for each mode. The following procedure was implemented in MATLAB R2015b (Mathworks, Inc, Natick, MA, USA). It used the packages SPLINEFIT [29] and spec file reader [30] developed for MATLAB and produced accurate mode estimations even with noisy data. First, the intensity histogram of each image was smoothed with robust locally weighted regression (robust loess, [31]) using a quadratic fitting model and a of span of 1% of all data points. After parameterisation via cubic spline fitting, the intensity values of the second derivative’s zero-crossings were determined. The intensity value within the intensity range (1000, 2500) corresponding to the GM mode and the intensity range (3000, 3500) corresponding to the WM mode were selected.

A transformation function was calculated by piecewise linear interpolation between the minimum intensity (I_Min_ = 0), the intensity levels of the GM and WM modes, and the maximum intensity (I_Max_ = 4095). The resulting transformation function bends at two anchor points corresponding to the intensities of the target and reference histogram modes and linearly matches the intensity values in between.

#### Non-Linear Histogram Matching (NHM)

PHM has the drawback that artificial contrast is introduced at the intensity levels around each anchor point of the transformation function, which affects, in our variant, the most frequent intensities in the MR image. To avoid this, we tested a smoothed variant by fitting a non-linear transformation function to the anchor points. To prevent overshoots and undershoots, the two anchor points of the PHM were complemented by two additional points along the coordinate system’s diagonal at intensities 1 and 4094. Using the SPLINEFIT package for MATLAB, a cubic spline was fitted to these 4 anchor points plus I_Min_ = 0 and I_Max_ = 4095 and used to remap the target image intensities.

#### Cumulative Histogram Matching (CHM)

As explained in the introduction, the matching of histogram percentiles can be carried to the extreme by matching the relative cumulative frequencies of the target image and the reference image at each single intensity level. Starting with the cumulative histograms of the target image and the reference image, a non-linear transformation function is sought with the constraint that it is monotonically increasing, thereby conserving the order from black to white in the grey scale. This function can be easily obtained by assigning to each intensity level *I*_*T*_ of the target image the intensity level of the reference image *I*_*R*_ that satisfies the condition that the corresponding relative cumulative frequencies *P*(*I*_*T*_) and *P*(*I*_*R*_) are equal or close to equal [22]. This algorithm is implemented in MIPAV. It initially classifies the data according to Izenman [32], which resulted in fewer than 200 different intensity levels in the corrected target images. We refined the classification to 1024 intensity levels.

#### ROI-Based Linear Standardisation (RLS)

**Fig 1:**
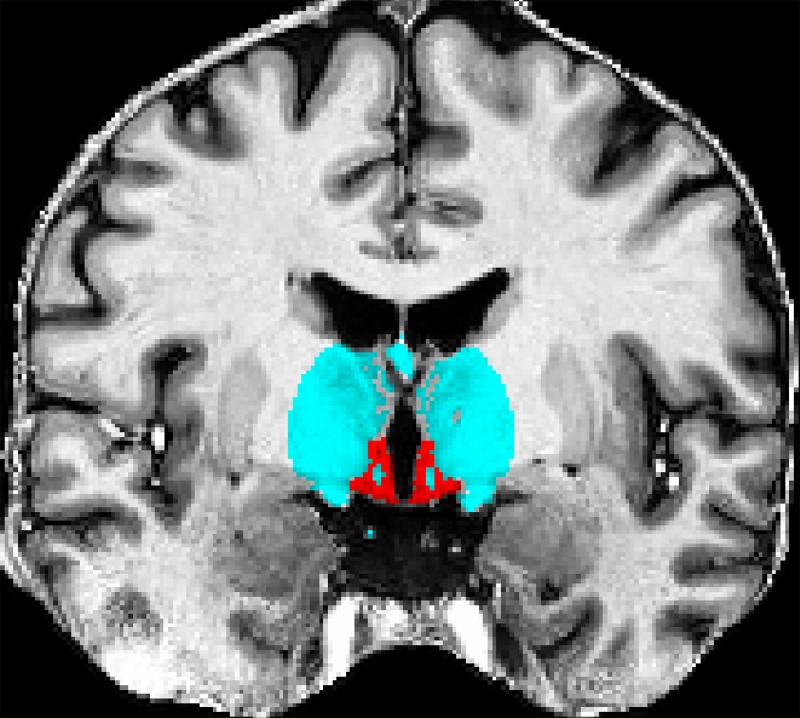
Coronal view of the hypothalamic GM ROI (red) and surrounding WM ROI (light blue) used for the RLS.

To estimate a simple transformation function that integrates both intensity and spatial information, we created individual ROIs for GM and WM (Fig 1) for each image. Then, the intensity relationship between target and reference ROIs was approximated with a linear model.

In detail, for the GM ROI a mask of the hypothalamus was created manually on the T1-weighted reference image following established guidelines [3]. To create comparable target-specific masks, we non-linearly registered this mask onto each native T1-weighted target image using the symmetric diffeomorphic image registration (SyN [33]) algorithm from the Advanced Normalization Tools (ANTs [34]) through a plugin for MIPAV. CSF voxels and WM voxels (e.g. fornix, vessels) were removed from the masks by using the tissue class maps from the FANTASM segmentation that had been performed to select the study sample (see chapter “Sample Selection Procedure”).

For the WM ROI, a spherical mask with a radius of 2 cm surrounding the hypothalamus but excluding the corpus callosum, was defined on the WM tissue map of the T1-weighted reference image. This mask was also non-linearly registered onto each target image and corrected for GM voxels and CSF voxels as defined by the tissue class maps from the FANTASM segmentation. Vessel voxels erroneously included in the reference WM ROI were thus mostly removed in the target WM ROIs. The WM ROI is dominated by voxels from the major WM tracts running through the hypothalamus (e.g. fornix, optic tract, and mamillary fasciculus) as well as WM surrounding the hypothalamus (e.g. anterior commissure, capsula interna, cerebral peduncle, thalamic WM, and high-intensity voxels of the basal ganglia).

With the median intensity values of the hypothalamic GM ROI and the surrounding WM ROI, a linear transformation function of the form ${\bar{I}}_{T}={\bar{I}}_{R}\cdot m+n$ was calculated and used to standardise the target images.

#### Segmentation-Based Piecewise Linear Standardisation (SPS)

To meet the demands of a potential tissue-specific scaling of image intensities we developed an intensity standardisation that evaluates both intensity and spatial information. The three major tissue classes—obtained from the FANTASM segmentation during sample selection—were analysed. A comparison of the tissue mean and median values within our sample revealed that the mean estimates were more homogeneous ($SD_{\bar{GM}}\;=\;75.9$; $SD_{\bar{WM}}\;=\;58.5$; $SD_{\bar{CSF}}\;=\;130.9$) than the median estimates ($SD_{\bar{GM}}\;=\;83.5$; $SD_{\bar{WM}}\;=\;68.2$; $SD_{\bar{CSF}}\;=\;188.0$). To match the mean intensities of each tissue class of the target image to those of the reference image, a continuous piecewise linear transformation function was calculated between I_Min_ = 0, the intensity levels of the three tissue means, and I_Max_ = 4095.

#### T1 mapping

In addition to the five intensity standardisation techniques for T1-weighted images, we included the quantitative T1 maps in our comparisons. As an alternative to T1-weighting, which reflects a mixture of parameters (T1, proton density, T2*), the MP2RAGE sequence provides quantitative measurements of the tissues’ variations in the T1 value. Being a reliable intrinsic parameter, its mapping on an absolute scale (time in milliseconds) should be comparable across subjects and scanners, that is, quantitative T1 maps should not require intensity standardisation and should not be subjected to any kind of correction. However, biological variations due to development in general, ageing, or disease [35–38] may pose problems for intensity-based analyses analogous to those experienced with T1-weighted images.

### Evaluation criteria and statistical procedures

#### Histogram comparison

After intensity standardisation, each target image was segmented into the three major tissue types (WM, GM, and CSF) with the FANTASM module in MIPAV (cf. chapter “Sample Selection Procedure”). Using the hard segmentation maps to identify GM and WM voxels, separate intensity histograms were computed for the whole-brain WM and GM areas of each intensity standardised target image. Standard error measures comparing the tissue-specific intensity histograms of the target images with that of the reference image would be biased against intensity standardisation techniques that produce histograms with empty intensity levels or local voxel accumulations at certain intensities. Such a bias is reduced in the cumulative probability distribution. The absolute error between the tissue-specific cumulative probability distribution of each target image and the reference image of the same contrast (T1-weighted; T1 map) was determined. The average of the absolute error within the shared intensity range was analysed as a global similarity measure between both distributions. In addition, the maximum absolute error was determined. Indicative of local histogram differences it can be considered an especially strict criterion.

Whole-brain measures can easily conceal local intensity variations. For a focused evaluation, we repeated this analysis within the target-specific GM ROIs and WM ROIs from the RLS. Though few, residual vessel voxels were manually removed from all GM and WM ROIs to rule out unwarranted effects due to their high intensities.

The average and maximum absolute errors of the native T1-weighted images were compared to those of the standardised T1-weighted images and the T1 maps, respectively, using two-tailed Wilcoxon Signed-ranks tests from SPSS statistics 18.0.0 (SPSS Inc. Released 2009. PASW Statistics for Windows. Chicago, USA). This test was chosen as a nonparametric alternative to the paired t-test to account for not normally distributed data (Shapiro-Wilk test, p > 0.05) and negative correlations between some of the compared data series. Lacking a global hypothesis that would be subject to alpha error accumulation, p-values below 0.05 were considered significant. The effect size r was computed from the standard normal deviate Z. In line with Cohen’s recommendations [39], values of r = 0.10, 0.30, and 0.50 were considered “small”, “medium”, and “large” effects, respectively.

#### Average image intensity

Comparing histograms alone can be misleading, because spatial correspondence between the image details contributing to the evaluated histogram characteristics cannot be verified. To compare the image intensities of the native and standardised images relative to their position in space, the target images (native and intensity standardised) were non-linearly registered onto the reference image. For this, the registration of the reference image to each target image, performed in the context of the RLS was inverted and applied to the target images with nearest neighbour interpolation. By this procedure, optimal spatial correspondence between each native and intensity standardised target image with the reference image was obtained. Likewise, the tissue class maps from the FANTASM segmentation (hard segmentation) of the native and intensity standardised images were co-registered onto the reference image. Then, the average voxelwise intensity difference between the reference image and the co-registered target image was computed for all voxels defined as WM by the tissue class maps of both the reference and the target image. Analogously, we computed the average voxelwise intensity difference for the whole-brain GM areas.

In addition, we determined the average voxelwise intensity difference for the hypothalamic GM ROI and surrounding WM ROI of the reference image from the RLS. Residual vessel voxels had already been removed from both ROIs during the histogram comparison.

Again, Wilcoxon Signed-ranks tests (two-tailed) were used to compare the differences of the native and intensity standardised T1-weighted images, accounting for not normally distributed data and negative correlations between some data series.

Voxels in quantitative T1 data sets reflect the tissue-specific longitudinal relaxation time in seconds. Small biological differences in T1 appear as low contrast between tissue types. Due to the lower contrast of the T1 maps, their absolute voxelwise intensity differences cannot become as large as those of the high-contrast T1-weighted images (whose contrast is related to the sequence parameter selection [40]). To ensure a correct comparison between both MR contrasts, the intensity differences would need to be normalised with an estimate of the intensity variation. Observing superiority *or* inferiority of the T1 maps over the T1-weighted images, depending on whether the range or the interquartile range was chosen for such a normalisation, we had to exclude the T1 maps from these analyses.

#### Local image intensity

For a detailed analysis of the image intensities, we examined the intensity of the boundary between GM and WM in the region of the hypothalamus. For this, the native and intensity standardised images were co-registered into a coordinate system compatible to the atlas by Mai et al. [41] using the Leipzig Image Processing and Statistical Inference Algorithms (LIPSIA [42]) with shifted linear interpolation [43]. By piecewise linear mapping from the greyscale intensity range (0, 4000) to the RGB colour space (3 x 8-bit), the T1-weighted images were colour-coded using three colours (cf. [3]). Red (255:0:0) was assigned to I_Min_ = 0% of the intensity range, blue (0:0:255) to I_Max_ = 100%, and the intermediary colour white (255:255:255) was adjustable. With sufficient anatomical expertise, the boundary between GM and WM and the corresponding intensity, respectively, can be easily identified by manipulating the intermediary colour white until it highlights the desired anatomical features. The imaging software ITK-SNAP [44] provides a graphical user interface for this purpose.

For the T1-weighted reference image, the boundary between hypothalamic GM and surrounding WM was found at 60% of the intensity range. For the reference’s T1 map the intensity range had to be adjusted to ensure a correct comparison. A comparable contrast and colour allocation was achieved by mapping intensities in the range (950, 3700) with the following assignments: blue: I_Min_ = 0%, white: I = 40%, and red: I_Max_ = 100%. With the reference’s intensity ranges as default, the rater had to determine the intensity of the boundary between hypothalamic GM and surrounding WM for each target image. (After perfect intensity standardisation, the boundary would have the same intensity across all target images). An experienced rater, who was blind to the intensity standardisation technique, performed two estimation runs which were then averaged. The test-retest reliability of this procedure was estimated with the intra-class correlation coefficient (ICC). It was very high for all standardisation techniques (ICC >.95). The intensity values of the hypothalamic boundary were normally distributed for each standardisation technique, warranting parametric variance comparisons for two correlated samples. The Pitman-Morgan test (two-tailed) was chosen to compare the inter-individual intensity variation of the hypothalamic boundary in the native T1-weighted images with the variation in the standardised T1-weighted images and the T1 maps, respectively.

#### Maintenance of biological variation

When correcting the image intensity, we have to make sure that only erroneous differences in the image intensity or T1-shifts are corrected, but volume-related intensity differences are maintained. Without external or longitudinal data it is difficult to disentangle these factors. We can approach this problem indirectly by proving that during intensity standardisation the relevant biological variance in certain volume measurements is preserved.

Effects for age have been described for T1 values in WM and GM [36, 38] as well as for tissue volume estimates (for reviews, see e.g. [45, 46, 47]). Given that age patterns exist in our volumetric data independently from T1-shifts and measurement errors, they should be preserved during intensity standardisation. To test this, the volumes of the whole-brain WM, GM, and CSF were calculated using the tissue class maps from the FANTASM segmentation of the native and intensity standardised images. The Pearson correlations between the whole-brain WM and GM volumes and age of the subjects were determined. Steiger’s equation [48] for the comparison of two dependent correlations was used to test for changes (two-tailed) of the correlation estimates following intensity standardisation. The magnitude of a correlation difference is given by Cohen’s effect size q; with q = 0.10, 0.30, and 0.50 considered “small”, “medium”, and “large” effect sizes, respectively [39].

The T1 maps were of special interest for this evaluation criterion. The quantitative mapping of the longitudinal relaxation time with the MP2RAGE sequence is advertised as being free of reception bias field, and, to a large extent, transmit field inhomogeneity [9]. Volumetric estimations based on quantitative T1 maps should therefore be free of technically induced differences in the T1 values. Inter-individual differences should be solely due to actual volume differences or T1 shifts affecting the volume estimation. Thus, T1 maps provide a validation for the correlation between tissue volumes and age within the native T1-weighted images.

## Results

### Histogram comparison

Both techniques that matched the histogram modes improved the homogeneity of the WM histograms at the cost of the homogeneity of the GM histograms: PHM improved the alignment of the target distribution with the reference distribution for the whole-brain WM area as indicated by significant reductions of the average absolute error and the maximum absolute error (large effects; Table 1). In the WM ROI it improved the average absolute error alone (medium effect). These improvements were accompanied by medium to strong *increases* in both error measures in the whole-brain GM and in the GM ROI, outlining an overall worsening of the GM histogram homogeneity (Table 2). Similarly, NHM significantly improved the histogram alignment for the whole-brain WM and the WM ROI but it *increased* the histogram divergence for the whole-brain GM and the GM ROI (medium and large effects).

**Table 1:** Results of the histogram comparisons for the whole-brain WM and the WM ROI. Improved intensity histogram alignment is represented by reduced average absolute errors and maximum absolute errors between the target and reference distributions of the intensity standardised T1-weighted images and of the T1 maps, respectively. The results of the Wilcoxon Signed-ranks tests (Z, p) and the effect size r are shown. CHM cumulative histogram matching, NHM non-linear histogram matching, PHM piecewise linear histogram matching, RLS ROI-based linear standardisation, ROI region of interest, SPS segmentation-based piecewise linear standardisation, WM white matter.

	WM average error										WM maximum error									
	Whole-brain WM					WM ROI					Whole-brain WM					WM ROI				
	Median	Range	Z	p	r	Median	Range	Z	p	r	Median	Range	Z	p	r	Median	Range	Z	p	r
T1-weighted	0.052	0.019–0.121				0.099	0.010–0.224				0.179	0.047–0.339				0.181	0.023–0.460			
PHM	0.022	0.005–0.031	3.73	1.9E-04***	0.59	0.064	0.026–0.116	2.46	0.014*	0.39	0.060	0.017–0.093	3.81	1.4E-04***	0.60	0.169	0.069–0.326	1.64	0.100	0.26
NHM	0.024	0.008–0.044	3.36	7.8E-04***	0.53	0.047	0.022–0.101	2.80	0.005**	0.44	0.063	0.020–0.103	3.77	1.6E-04***	0.60	0.114	0.056–0.274	2.35	0.019*	0.37
CHM	0.002	0.001–0.003	3.92	8.9E-05***	0.62	0.066	0.027–0.134	1.57	0.117	0.25	0.005	0.004–0.013	3.92	8.9E-05***	0.62	0.213	0.077–0.348	-0.16	0.872	-0.03
RLS	0.042	0.015–0.143	0.82	0.411	0.13	0.042	0.017–0.092	3.14	0.002**	0.50	0.103	0.030–0.294	2.50	0.012*	0.40	0.094	0.037–0.198	3.14	0.002**	0.50
SPS	0.016	0.007–0.030	3.88	1.0E-04***	0.61	0.048	0.017–0.142	3.10	0.002**	0.49	0.058	0.032–0.125	3.88	1.0E-04***	0.61	0.116	0.034–0.316	2.95	0.003**	0.47
T1 map	0.039	0.018–0.090	3.10	0.002**	0.49	0.083	0.011–0.181	3.62	2.9E-04***	0.57	0.154	0.079–0.326	1.75	0.079	0.28	0.178	0.026–0.404	0.60	0.550	0.09

* p < 0.05,

** p < 0.01,

*** p < 0.001.

**Table 2:** Results of the histogram comparisons for the whole-brain GM and the GM ROI. Improved intensity histogram alignment is represented by reduced average absolute errors and maximum absolute errors between the target and reference distributions of the intensity standardised T1-weighted images and of the T1 maps, respectively. The results of the Wilcoxon Signed-ranks tests (Z, p) and the effect size r are shown. CHM cumulative histogram matching, GM grey matter, NHM non-linear histogram matching, PHM piecewise linear histogram matching, RLS ROI-based linear standardisation, ROI region of interest, SPS segmentation-based piecewise linear standardisation.

	GM average error										GM maximum error									
	Whole-Brain					ROI					Whole-Brain					ROI				
	Median	Range	Z	p	r	Median	Range	Z	p	r	Median	Range	Z	p	r	Median	Range	Z	p	r
T1-weighted	0.058	0.013–0.113				0.082	0.024–0.172				0.088	0.018–0.119				0.142	0.055–0.289			
PHM	0.109	0.006–0.209	-3.21	1.3E-03**	-0.51	0.178	0.024–0.332	-2.58	0.010**	-0.41	0.143	0.015–0.288	-3.55	3.9E-04***	-0.56	0.299	0.050–0.503	-2.50	0.012*	-0.40
NHM	0.139	0.020–0.258	-3.62	2.9E-04***	-0.57	0.191	0.023–0.368	-2.69	0.007**	-0.43	0.180	0.034–0.322	-3.66	2.5E-04***	-0.58	0.304	0.056–0.536	-2.43	0.015*	-0.38
CHM	0.001	0.001–0.059	3.66	2.5E-04***	0.58	0.055	0.013–0.176	0.97	0.332	0.15	0.004	0.002–0.119	3.58	3.4E-04***	0.57	0.125	0.046–0.363	0.49	0.627	0.08
RLS	0.045	0.021–0.180	0.47	0.641	0.07	0.032	0.019–0.053	3.81	1.4E-04***	0.60	0.094	0.030–0.215	-1.74	0.083	-0.27	0.083	0.065–0.141	3.73	1.9E-04***	0.59
SPS	0.012	0.002–0.039	3.78	1.6E-04***	0.60	0.049	0.022–0.123	3.06	0.002**	0.48	0.027	0.006–0.060	3.81	1.4E-04***	0.60	0.100	0.047–0.239	3.19	1.4E-03**	0.50
T1 map	0.044	0.015–0.119	0.11	0.911	0.02	0.084	0.024–0.155	0.37	0.709	0.06	0.067	0.024–0.163	1.01	0.313	0.16	0.153	0.056–0.260	-0.82	0,411	-0.13

* p < 0.05,

** p < 0.01,

*** p < 0.001.

While CHM improved the histogram alignment in the whole-brain analyses RLS showed best performance in the ROI analyses. The former significantly reduced both error measures for the whole-brain WM and GM areas (large effects) but yielded no significant change at all within the two ROIs. Conversely, RLS produced large-sized reductions for both error measures within the WM ROI and the GM ROI and yielded no improvements of the whole-brain tissue-specific histograms except for a medium-sized reduction of the maximum absolute error in the WM.

SPS reduced the average absolute error and the maximum absolute error in both tissue classes, in the whole-brain analyses (large effects) as well as in the ROI analyses (medium to large effects).

The quantitative T1 maps appeared not as homogeneous across subjects as expected. The average absolute error between the target T1 map distributions and the reference T1 map distribution was significantly smaller than that of the T1-weighted images, both in the whole-brain WM and in the WM ROI (medium and large effect). The maximum absolute error—an indicator for local deviations and thus a stricter criterion—was not reduced though. In addition, in all GM histogram comparisons the T1 maps were statistically indistinguishable from the T1-weighted images.

### Average image intensity

Consistent results were obtained for the whole-brain analyses versus the ROI analyses of the average image intensity but, again, a distinction between the two tissue classes GM and WM is vital. The average voxelwise intensity difference between the T1-weighted reference image and the native T1-weighted target images had a sample median (range) of 251.7 (226.2–292.9) in the whole-brain WM and a median of 259.3 (192.7–343.9) in the WM ROI. These differences were significantly reduced after intensity standardisation with any of the five methods (Table 3). The effect sizes were large except for a medium effect (r = .49) observed after SPS in the WM ROI.

**Table 3:** Reductions of the average voxelwise intensity difference following intensity standardisation. The results of the Wilcoxon Signed-ranks tests (Z, p) and the effect size r are shown. CHM cumulative histogram matching, GM grey matter, n/a not applicable, NHM non-linear histogram matching, PHM piecewise linear histogram matching, RLS ROI-based linear standardisation, ROI region of interest, SPS segmentation-based piecewise linear standardisation, WM white matter.

	WM Intensity Difference										GM Intensity Difference									
	Whole-Brain					ROI					Whole-Brain					ROI				
	Median	Range	Z	p	r	Median	Range	Z	p	r	Median	Range	Z	p	r	Median	Range	Z	P	r
T1-weighted	251.7	226.2–292.9				259.3	192.7–343.9				419.4	408.4–443.6				440.0	341.3–606.8			
PHM	219.9	206.4–254.1	3.88	1.0E-04***	0.61	194.9	175.6–310.8	3.92	8.9E-05***	0.62	430.1	410.5–465.9	-2.02	0.044*	-0.32	493.6	351.7–562.5	-1.05	0.296	-0.17
NHM	223.8	210.3–258.1	3.85	1.2E-04***	0.61	196.7	173.7–319.3	3.92	8.9E-05***	0.62	435.1	409.3–482.7	-2.17	0.030*	-0.34	474.1	345.2–547.4	-0.34	0.737	-0.05
CHM	230.0	221.4–258.9	3.92	8.9E-05***	0.62	216.5	185.0–276.7	3.14	0.002**	0.50	418.7	408.3–433.1	0.07	0.940	0.01	432.6	352.7–574.1	0.60	0.550	0.09
RLS	226.4	210.0–252.7	3.85	1.2E-04***	0.61	189.4	178.1–282.6	3.92	8.9E-05***	0.62	400.8	384.2–436.8	3.66	2.5E-04***	0.58	362.3	324.1–514.2	3.92	8.9E-05***	0.62
SPS	234.0	217.9–266.7	3.73	1.9E-04***	0.59	213.8	186.6–352.9	3.10	0.002**	0.49	412.5	407.8–432.6	3.47	5.2E-04***	0.55	387.7	352.7–582.5	3.66	2.5E-04***	0.58
T1 map	n/a	n/a	n/a	n/a	n/a	n/a	n/a	n/a	n/a	n/a	n/a	n/a	n/a	n/a	n/a	n/a	n/a	n/a	n/a	n/a

* p < 0.05,

** p < 0.01,

*** p < 0.001.

In the whole-brain GM the median of the average voxelwise intensity difference was 419.4 (408.4–443.6) and in the GM ROI the median was 440.0 (341.3–606.8). Continuing their pattern from the histogram comparison, both mode-based histogram matchings (PHM and NHM) *increased* the average voxelwise intensity difference in the GM, which became significant in the whole-brain analysis. CHM did not improve the GM intensities in the whole-brain image or in the ROI. In contrast, consistent improvements (medium to large effects) were obtained after RLS and SPS.

### Local image intensity

**Fig 2:**
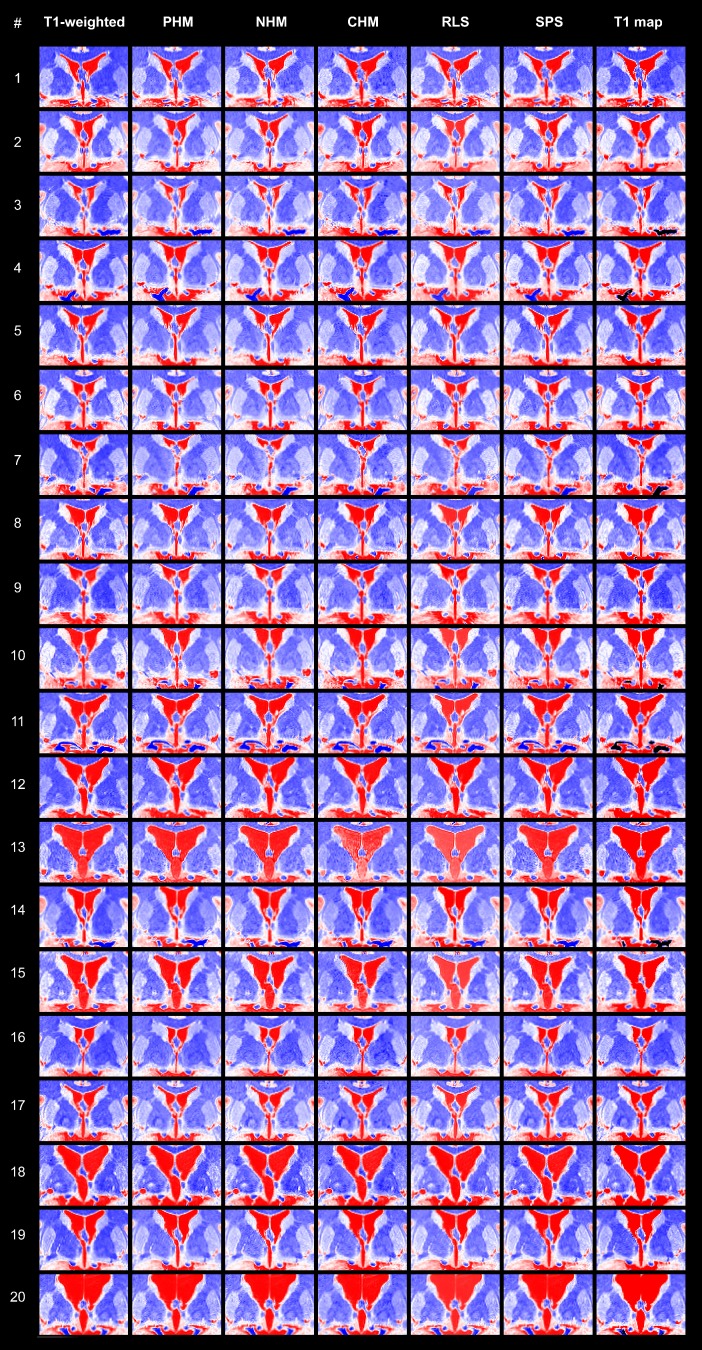
Inter-individual intensity variation revealed by colour-coding. Coronal view of all target images—the two MP2RAGE contrasts (column 1 and 7) and the intensity standardisation results (columns 2–6). Compare, e.g. #9 with overexposed GM and WM, and #10 with relatively dark GM and WM. CHM cumulative histogram matching, NHM non-linear histogram matching, PHM piecewise linear histogram matching, RLS ROI-based linear standardisation, SPS segmentation-based piecewise linear standardisation.

**Fig 3:**
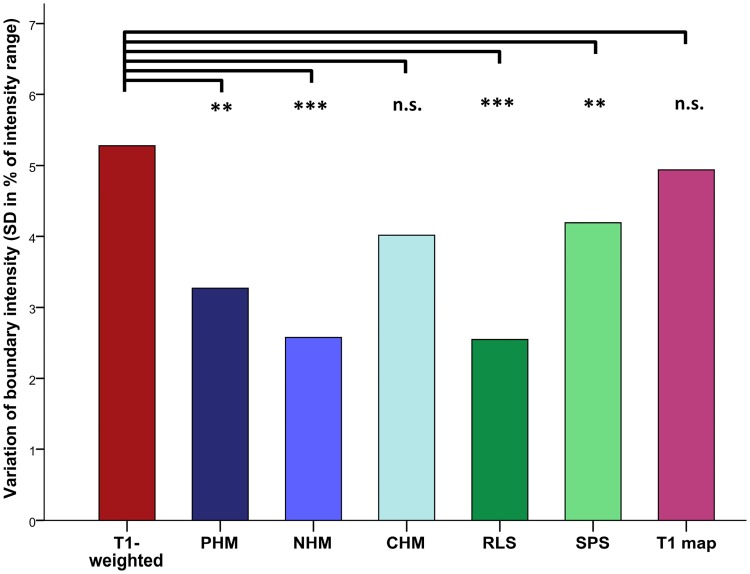
Inter-individual intensity variation of the hypothalamus boundary. The results of the Pitman-Morgan tests comparing the variation of the native T1-weighted images with the standardised images and the T1 maps, respectively, are shown. CHM cumulative histogram matching, NHM non-linear histogram matching, n.s. not significant, PHM piecewise linear histogram matching, RLS ROI-based linear standardisation, SD standard deviation, SPS segmentation-based piecewise linear standardisation, ** p < 0.01, *** p< 0.001.

Fig 2 shows the colour-coded images before (column 1) and after intensity standardisation (columns 2–6), as well as the T1 maps (column 7). The colour-mappings developed for the reference T1-weighted image and the reference T1 map (see Methods chapter “Local image intensity”) were applied. Intensity differences between the brains are prominent in local GM regions (e.g. amygdala and hypothalamus; optimally in red) and also in the overall impression, which is dominated by the WM (blue). In the native T1-weighted target images, the intensity of the boundary (in % of the intensity range) between hypothalamic GM and surrounding WM varied with a SD of 5.3, which corresponds to 211 intensity levels. The variation was significantly reduced by PHM (SD = 3.3, t(18) = 3.89, p = 0.001) and NHM (SD = 2.6, t(18) = 4.97, p < 0.001, Fig 3). CHM failed to satisfy this criterion (SD = 4.0, t(18) = 1.55, p = 0.139). After RLS, we observed the smallest inter-individual intensity variation of the hypothalamic boundary (SD = 2.5, t(18) = 7.18, p < 0.001). Considerably weaker changes, although still significant, were achieved by SPS (SD = 4.2, t(18) = 3.66, p = 0.002). Remarkably, the intensity variation of the hypothalamic boundary in the T1 maps was found to be of similar magnitude compared to the T1-weighted images when the T1 maps were presented with a comparable contrast to the rater (SD = 4.9, t(18) = 1.30, p = 0.221).

### Maintenance of biological variation

**Fig 4:**
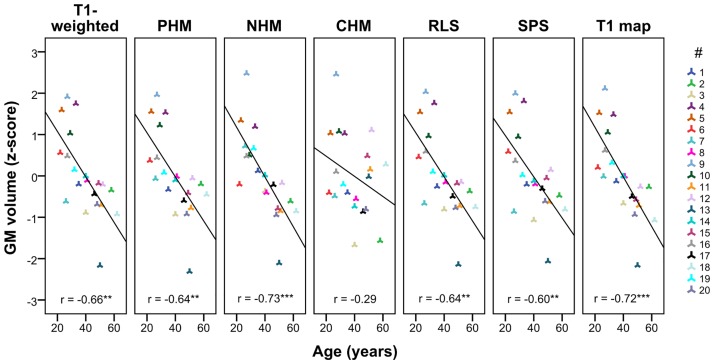
Correlations (r) between subject (#) age and whole-brain GM volumes. CHM (4^th^ column from right) drastically reduced the correlation between age and GM volume originally observed in the native T1-weighted images (1^st^ column). L linear, NL non-linear, PWL piecewise linear, ** p < 0.01, *** p < 0.001.

Before intensity standardisation there was no significant correlation between the WM volume in the native T1-weighted images and subject age, but the GM volume showed a medium negative correlation with subject age (r = -0.66, p = 0.001). This correlation was replicated by the intensity standardisation techniques and the T1 maps (Fig 4) except for CHM (Z = -2.17; p = 0.030) and SPS (Z = -2.60; p = 0.009). Whereas the latter caused only a small-sized drop of this correlation (Cohen’s *q* = 0.11), CHM introduced severe changes to the volumetric pattern (Cohen’s *q* = 0.50, large effect size). Further analysis of this technique suggested that this drastic change was a consequence of imposing the reference’s GM/WM volume ratio onto each target image.

## Interpretation and conclusions

Intensity-based MR image analysis requires that similar image intensity values represent similar tissue types across different persons. By applying colour-coding to T1-weighted MP2RAGE images, we found that the high measurement accuracy achieved by high-resolution imaging might be compromised by inter-individual image intensity variation. Because investigating the source of this variance was beyond the scope of this study, we tested the performance of a variety of intensity correction procedures. We included histogram-based techniques as well as methods that evaluate both intensity and spatial information. A mapping of the longitudinal relaxation time was included as well, as, in theory, it should be free of artificial intensity variation. To test the performance with severe cases, the sample consisted of images with extreme intensities in the GM and WM that were matched to a representative reference image.

A multi-level evaluation was performed with a focus on the hypothalamic region. We assessed the alignment of the cumulative probability distributions under the assumption that images with similar intensity histograms look more alike than images with divergent intensity histograms. This was done separately for the whole-brain GM and WM areas, as well as for a GM ROI of the hypothalamus and a ROI of WM surrounding the hypothalamus. Additionally, the average voxelwise intensity difference between the reference image and the co-registered target images was evaluated in the ROIs and the whole-brain tissues. We semi-automatically determined the intensity of a selected anatomical detail—the hypothalamic boundary—and analysed its variability before versus after intensity standardisation. Finally, we required that the original biological information within the native T1-weighted images be preserved during intensity standardisation. This was measured by the correlation between whole-brain tissue volumes and subject age. Comparable correlations observed in the quantitative values of the T1 maps confirmed the legitimacy of this demand.

### Standardisations evaluating intensity domain and spatial domain

#### ROI-based linear standardisation

A global linear scaling factor estimated from precise subcortical GM and WM ROIs performed best among the tested methods: it improved the histogram alignment within both ROIs which was indicated by significantly reduced average absolute errors and maximum absolute errors (large effects). The whole-brain histograms of GM and WM tissue areas remained mostly untouched, though. Nonetheless, and possibly more important for its applicability, the average voxelwise intensity differences between reference image and target images were significantly reduced, both in the ROIs and in the whole-brain tissues (large effect sizes). Furthermore, RLS reduced (SD = 2.5) the original inter-individual intensity variation (SD = 5.3) of the hypothalamus boundary by more than half. This means, after standardisation with RLS, the hypothalamic boundary can be detected at the same intensity across different subjects. The increased inter-individual homogeneity of the intensity of the hypothalamic boundary facilitates the development of automated segmentation methods greatly as they can rely more strongly on the image intensities. The computationally expensive creation of an atlas (e.g. probability map) is unnecessary. Finally, RLS preserved the original biological information within the images, as indicated by a stable correlation between the whole-brain GM volume and subject age.

#### Segmentation-based piecewise linear standardisation

With SPS, we aimed at integrating spatial and intensity information to obtain a tissue-specific scaling of the image intensities. In the histogram comparisons, we achieved consistent improvements of both error measures for the two whole-brain tissues (large effects) as well as for the two ROIs (medium to large effects). These were accompanied by medium- to strong-sized improvements of the average image intensity for all four analysed regions. The reductions in the intensity variation of the hypothalamic boundary were considerably less pronounced (SD = 4.2) than with RLS and they came at the cost of a small-sized, significant reduction of the correlation between the whole-brain GM volume and subject age.

### Standardisation techniques evaluating only the intensity domain

#### Piecewise linear histogram matching and non-linear histogram matching

MP2RAGE histograms show distinct modes corresponding to the most frequent GM and WM intensities. Matching these modes by piecewise linear interpolation (PHM) or with a non-linear correction function (NHM) mostly improved the alignment of the whole-brain WM histograms and the histograms of the WM ROI. However, for the whole-brain GM and the GM ROI, significant, consistent *increases* of the histogram divergence became evident. We observed the same pattern at the analysis level of the actual images. Strong reductions were obtained for the voxelwise average intensity differences in the WM while the differences were unchanged or increased in the GM. When looking at the agreement of both criteria, we conclude that the GM mode is a suboptimal histogram characteristic for T1-weighted MP2RAGE images. This is possibly due to the wide spread of the GM histogram mode. Nonetheless, substantial reductions of the intensity variation of the hypothalamic boundary were obtained with PHM and NHM (SD = 3.3, SD = 2.6, respectively), suggesting that this criterion is related to an effective intensity correction in the WM. The original age-related pattern in the whole-brain GM volume was preserved by both techniques.

#### Cumulative histogram matching

CHM provided by the MIPAV software was strong in the histogram comparisons of the whole-brain tissues (large effects) but failed to affect the histograms of the subcortical ROIs (p > 0.05). Remarkably unrelated, the algorithm improved the average voxelwise intensity of the WM in the whole-brain image as well as in the ROI (large effect sizes), but the GM remained unchanged in both analysed regions. The variance reduction of the intensity of the hypothalamic boundary (SD = 4.0) did not reach significance level. There is no obvious reason as to why our evaluation criteria diverge after CHM. It might be a side effect of the drastic modifications forced onto the images by this method. CHM is closely related to methods that match histogram percentiles or the histogram median, respectively. It assumes that, up to a certain intensity level, the target image has accumulated the same relative number of voxels as the reference image. This is illustrated by the fact that the reference’s GM/WM volume ratio was imposed onto each target image. This might be appropriate for selected purposes like matching histological images to MR images of the same subject [24], but it certainly is not suited for inter-subject intensity matching when structural differences are of interest. Our fourth criterion substantiates this. The correlation between the whole-brain GM volume and subject age was strongly reduced, proving that relevant biological variance was lost. We conclude that CHM, like the matching of percentiles, will enforce similarity irrespective of tissue membership.

#### T1 mapping

Finally, our comparison included quantitative T1 maps. Reflecting the tissues’ variations in the T1 relaxation time, which is a reliable intrinsic parameter, on an absolute scale (time in seconds), they should not require intensity standardisation. In the histogram comparisons, only the average absolute error substantiated the T1 maps’ theoretical superiority over the T1-weighted images; and only for the WM (medium to large effects) but not for the GM. The maximum absolute error, indicative of local deviations and thus considered a stricter criterion, was not reduced in any of the analysed regions. Due to the lower contrast of the T1 maps, a conclusive comparison of the voxelwise intensity difference with that of the T1-weighted images was not possible. However, when the images were presented with a comparable contrast to the rater, the T1 maps evinced nearly the same colour allocation and intensity variation of the hypothalamus boundary, respectively (SD = 4.9), as the native T1-weighted images. The correlations of the whole-brain GM volumes with subject age were of the same height as those of the native T1-weighted images, suggesting that both MRI contrasts provide comparably good insights into volumetric patterns of this tissue class.

The inter-individual intensity differences of the hypothalamic boundary in the T1 maps were unexpected. Their magnitude was large enough to hinder manual segmentation of the hypothalamus. The images analysed in this study were acquired in the years 2010–2014 according to Marques et al. [9]. In 2013, Marques and Gruetter reported that transmit field (B_1_^+^) inhomogeneity correction improves the T1 estimation with the MP2RAGE sequence [40]. They quantified the error on the T1 estimation for a protocol that is similar to ours regarding flip angles and resolution, that is, sensitivity to B_1_^+^ inhomogeneity (protocol iia). The estimated error was less than 2.5% for the T1 value of WM (1.2s [49]) when the B_1_^+^ field varies by ±40%–the typical range of B_1_^+^ in the human brain at 7T. This corresponds to a maximum error on the T1 values of WM of approximately 30 ms. It is not possible to estimate the transmit field post hoc. Any corrections that we could do now, without knowledge of the exact distribution of the B_1_^+^ field, would change the T1-values in such a way that they would lose their meaning as a physical parameter (longitudinal relaxation time). To answer the question in how far a B_1_^+^ correction might have helped reducing the intensity variation in the T1 maps, we determined the location of the WM peak in the T1 map histograms of our sample. The T1 values of the peaks ranged from 1344 to 1499. The difference (155ms) is considerably larger than the B_1_^+^-related errors on the T1 estimation of WM observed by Marques and Gruetter. B1 inhomogeneity correction might have reduced the inter-individual intensity differences observed in the T1 maps of our study but a large portion would have remained.

### Limitations

We expect our results to be valid for the following conditions: firstly, the tested intensity standardisations followed the assumption that similar intensities represent similar tissue types across different images, that is, GM in one brain image should be as bright as GM in another brain image. In line with this, intensity variations were removed irrespective of their potential sources—be they technical in nature or biological like T1 tissue signal shifts. Secondly, the initial intensity differences between the 7T T1-weighted MP2RAGE images were moderate compared with standard MR sequences. We mitigated this possible limitation by selecting target images with extreme intensities in the GM and WM at the expense of generalisation to random image samples. Thirdly, the sequence parameters selected for the MP2RAGE provided optimum contrast-to-noise ratio between GM, WM, and CSF. The total acquisition time of 18:02 min was rather long for clinical practice and can easily be reduced, however, likely at the expense of CNR, SNR, or the field of view (cf. [9]). Moreover, the histograms of the T1-weighted images show distinct modes for GM and WM. With other sequences or lower field strengths, appropriate adaptations of our standardisation techniques may yield different results. For example, piecewise linear matching of histogram modes has been shown to be effective with unimodal intensity histograms [12] but failed with our multimodal histograms. Nonetheless, we expect the winning algorithm RLS to be robust with other sequences and field strengths if the ROIs are carefully chosen: a strong contrast between them will ensure a reliable transformation function, but intensities at the very ends of the intensity spectrum (e.g. CSF and vessel voxels) might contain little valid information. Bright WM and dark GM will therefore be optimal. Either regions are chosen whose MRI intensities are stable in age and disease, or further preprocessing steps ensure that abnormal tissue constitutes only a negligible portion that will not affect the median estimates of the ROIs. In the T1-weighted MP2RAGE images, we sometimes observed idiopathic hypointensities of the globus pallidum. With the help of the FANTASM tissue class maps, they were effectively removed from our WM ROI. Naturally, the two ROIs need to be large enough to provide a reliable median intensity estimate each, even after co-registration and cleansing. With roughly 2000 voxels our GM ROI should be considered the lower limit.

### Summary

RLS showed the best results for standardising intensities in T1-weighted MRI data sets. It fulfilled all evaluation criteria except for a weak performance with the whole-brain histograms. The algorithm is easily implemented and the ROI definition is facilitated by freely available registration and segmentation software.
